# Accuracy of perfusion MRI with high spatial but low temporal resolution to assess invasive breast cancer response to neoadjuvant chemotherapy: a retrospective study

**DOI:** 10.1186/1471-2407-11-361

**Published:** 2011-08-19

**Authors:** Cédric de Bazelaire, Raphael Calmon, Isabelle Thomassin, Clément Brunon, Anne-Sophie Hamy, Laure Fournier, Daniel Balvay, Marc Espié, Nathalie Siauve, Olivier Clément, Eric de Kerviler, Charles-André Cuénod

**Affiliations:** 1 Radiologie, Hôpital Saint-Louis - Inserm U728 - Université Paris VII, 1 Avenue Claude Vellefaux, Paris, 75010, France; 2 Radiologie, Hôpital Tenon - Inserm U970 - Université Paris VI, 4 rue de la chine, Paris, 75020, France; 3 Radiologie, Hôpital Européen George Pompidou - Inserm U970 - Université Paris V, 56 Rue Leblanc, Paris, 75015, France; 4 Centre des maladies du sein, Hôpital Saint-Louis - Université Paris 7, 1 Avenue Claude Vellefaux, Paris, 75015, France

## Abstract

**Background:**

To illustrate that Breast-MRI performed in high spatial resolution and low temporal resolution (1 minute) allows the measurement of kinetic parameters that can assess the final pathologic response to neoadjuvant chemotherapy in breast cancer.

**Methods:**

Breast-MRI was performed in 24 women before and after treatment. Eight series of 1.11 minute-duration were acquired with a sub-millimeter spatial resolution. Transfer constant (K^trans^) and leakage space (V_e_) were calculated using measured and theoretical Arterial Input Function (AIF). Changes in kinetic parameters after treatment obtained with both AIFs were compared with final pathologic response graded in non-responder (< 50% therapeutic effect), partial-responder (> 50% therapeutic effect) and complete responder. Accuracies to identify non-responders were compared with receiver operating characteristic curves.

**Results:**

With measured-AIF, changes in kinetic parameters measured after treatment were in agreement with the final pathological response. Changes in V_e _and K^trans ^were significantly different between non-(N = 11), partial-(N = 7), and complete (N = 6) responders, (P = 0.0092 and P = 0.0398 respectively). A decrease in V_e _of more than -72% and more than -84% for K^trans ^resulted in 73% sensitivity for identifying non-responders (specificity 92% and 77% respectively). A decrease in V_e _of more than -87% helped to identify complete responders (Sensitivity 89%, Specificity 83%). With theoretical-AIF, changes in kinetic parameters had lower accuracy.

**Conclusion:**

There is a good agreement between pathological findings and changes in kinetic parameters obtained with breast-MRI in high spatial and low temporal resolution when measured-AIF is used. Further studies are necessary to confirm whether MRI contrast kinetic parameters can be used earlier as a response predictor to neoadjuvant chemotherapy.

## Background

Neoadjuvant chemotherapy is increasingly used in breast cancer patients to decrease the tumour size in large cancers to enable breast-conserving treatment. Accurate evaluation of the treatment response before surgery offers the potential to avoid unnecessary mutilating procedures in patients with a favorable prognosis, without jeopardizing local control or long-term survival. Compared with physical examination, and conventional modalities (US and mammography), breast MRI appears to be the best monitoring method for neoadjuvant chemotherapy [1,2]. Although MR imaging may be superior to other methods [3,4], the correlation between conventional anatomic MRI analysis and histopathological response is not perfect [5]. The determination of residual tumour size is underestimated and unreliable in carcinomas significantly responding to chemotherapy which may lead to missed detections in up to 30% of patients [6]. There is now increasing evidence that functional analysis of the microcirculation by using dynamic contrast material-enhanced MR imaging could be used to identify responders and non-responders during and/or after neoadjuvant chemotherapy more reliably than conventional anatomic MRI results alone [6-10]. The functional analysis is based on post-therapeutic changes of microvessels permeability, tissue perfusion, blood volume, and extracellular leakage space. These parameters can be obtained by analyzing the enhancement kinetics measured in the tissue of interest and in its afferent artery (Arterial Input Function, AIF) using compartmental modeling [11,12].

Several compartmental models can be chosen for assessment. The simplest model, applied to MRI data by both Larsson et al. [13] and Tofts and Kermode [14] allows calculation of the transfer constant (K^trans^) that reflects simultaneously perfusion and permeability, and the leakage space (V_e_). More recent models potentially enable to distinguish perfusion and permeability separately [15-18]. The choice of the model and kinetic parameters to be calculated depends on the acquisition parameters. In particular, the temporal resolution determines whether the vascular component can be taken into account. Indeed, perfusion and blood volume measurement requires a high temporal resolution, because the sampling interval must be less than the mean transit time of the contrast agent [19], which is usually less than 2 seconds [20]. In breast MRI, high spatial resolution is required because most of the diagnostic criteria are based on lesion morphology [21]. Due to technical reasons in most current systems of magnetic field up to 1.5T, the 3D high spatial resolution limits the temporal resolution to as low as 1 minute and imposes to use the simplest model limited to the estimation of K^trans ^and V_e _values [22-24].

The estimation of the kinetic parameters requires two sets of data: the variation of contrast concentration in time in the tissue of interest and in the feeding artery (arterial input function, AIF). In MRI, concentrations can be non-invasively derived from signal intensity. In Breast MRI, the measurement of the AIF can be difficult. A large blood vessel such as the aorta is rarely included in the field of view [25] and the measurements of the internal thoracic artery can be difficult due to its small size with the risk of partial volume artifact [26]. For these reasons, several authors use a calculated theoretical AIF [7]. The time curve of contrast agent concentration in the plasma is represented by a biexponential decay based on the AIF measured by Weinmann et al [27]. However, the use of a calculated theoretical AIF may entail errors in MR estimates of kinetic parameters.

The purpose of this retrospective study is to demonstrate that even with low temporal resolution routine MRI protocol, changes in microcirculation kinetic parameters such as K^trans ^and V_e _can be used to determine tumor response to neoadjuvant chemotherapy in breast cancer as observed at the final pathological evaluation after surgery.

## Methods

### Demography

This study was part of the Remagus [28,29] protocol, approved by the ethics committee, and requiring informed consent before enrollment from all patients. Three physicians specialized in breast cancer with over 10 years of experience performed the patients follow up and determined the clinical response to neoadjuvant chemotherapy according the WHO criteria. The Remagus protocol was a neoadjuvant chemotherapy trial for locally advanced breast cancers including 4 cycles of Antracycline and Cyclophosphamide, followed by 4 cycles of Docetaxel chemotherapy. Patients with noninflammatory, stage II to III breast cancer were included during 12 month. Diagnosis of invasive breast carcinoma was made by core needle biopsy in all patients. Surgery was performed less than 4 weeks after the last course of chemotherapy. Patients either underwent mastectomy or wide local excision with axillary lymph node dissection.

### Pathologic Assessment

One senior pathologist with 20 years of experience in breast pathology blinded to the MRI results assessed tumor response and graded according to the scale established by Sataloff [30] as shown Table 1: total or near-total therapeutic effect (grade A), more than 50% therapeutic effect but less than total or near-total effect (grade B), less than 50% therapeutic effect but visible effect (grade C), or no therapeutic effect (grade D). Pathologic tumor regression was used as the gold standard to evaluate treatment response. For comparison with imaging five groups of patients were defined according to the response grade: complete responder group (grade A), partial responder group (grade B), responder group (A+B), non-responder group (grade C+D), and non-complete responders (grades B+C+D).

**Table 1:** Tumor response graded according to the scale established by Sataloff

Sataloff Grade	Therapeutic effect at pathology
**A**	Total or near-total
**B**	More than 50% therapeutic effect but less than total or near-total effect
**C**	Less than 50% therapeutic effect but visible effect
**D**	No therapeutic effect

### MRI protocol

All patients underwent two breast MRI, the first exam less than 1 week before the beginning of the treatment and the second MRI performed after completion of chemotherapy treatment and less than 2 weeks before surgery. All MRI were performed on a 1.5 Tesla Siemens Symphony TIM MR system (Erlangen, Germany), with a breast-specific coil with four elements, CP Breast Array Coil. DCE-MRI was acquired with a 3D T1-weighted gradient echo sequence using a TR of 4.67 ms, a TE of 1.65 ms and a flip angle of 12°. The DCE-MRI sequences (120 contiguous 1.2-mm-thick slices, 320 × 280 mm FOV, 380 × 300 matrix, 1.11 min scan duration, axial slices on both breasts) were acquired at 0, 1.36, 2.47, 3.59, 5.10, 6.21, 7.32, and 8.44 min. The contrast agent, gadoterate dimeglumine (Dotarem^®^, Guerbet, France), was injected immediately after the acquisition of the first DCE-MRI sequence with an automatic injector (Spectris, Medrad, UK) at a dose of 0.1 mmol/Kg with a rate of 2 ml/sec and pushed by saline serum. The minimum delay between the end of the contrast injection and the beginning of next sequence acquisition was 20 second.

### Data processing

A senior and a junior radiologist with 8 years and 2 years of experience in breast imaging respectively, performed all image analysis. Tumor sizes were measured with electronic calipers on high-resolution T1 weighted post contrast images (measured on the 90-120 second subtraction image), following the WHO methodology, the same day of the exam by the same radiologist as required by the clinical protocol [31], the functional analysis was performed retrospectively by the same radiologists. For this analysis, the use of a 3D acquisition for the DCE-MRI allowed the selection of region of interest (ROI) at a different level for tumor than for internal thoracic artery when necessary. Regions of interest were drawn by the radiologist on subtraction images with an appropriate window and magnification factor to optimize the detection of both enhancing tumor margin and internal thoracic artery. For the AIF, the center of the ROIs were manually defined in the center of the internal thoracic artery in the native images at the second acquisition time (first acquisition after injection), and the 8 surrounding pixels were automatically selected by the computer. This 9 pixel square was automatically reproduced at the same position at all acquisition time. Then, each ROI was manually translated when necessary for motion artifacts correction to avoid partial volume artifacts. The tumor ROIs were placed around the edge of the anatomically defined tumor but away from non-enhancing areas which were either necrotic or so poorly perfused that they could not be evaluated in functional imaging.

With the 3D T1-weighted gradient echo sequence use for the DCE-MRI, the contrast information was recorded 30 seconds after the beginning of the acquisition and at least 50 second after the injection. Thus, we assumed that the contrast information was measured after the first pass of the bolus of contrast media in the artery and therefore after the peak concentration [32]. Hence, for all patients, contrast media concentrations in artery and tumor were supposed to be low (< 2 mM Gd-DOTA) and a linear relationship was assumed between signal intensity kinetics measured in ROIs and contrast media concentrations according with the following equations:

(1) $$C_{t}\left(t\right)=R\cdot \left(IS_{t}\left(t\right)-IS_{t}\left(0\right)\right)$$

(2) $$C_{p}\left(t\right)=R\cdot \left(IS_{p}\left(t\right)-IS_{p}\left(0\right)\right)$$

Where C_t _and C_p _are the concentrations of contrast media in the tissue of interest and plasma, respectively. IS_t _and IS_p _are the signal intensities measured with the ROIs in the tissue of interest and the internal thoracic artery, respectively. R is an amplitude constant, which is simplified in equation 3.

Custom software [33] written with Matlab^® ^(MathWorks Inc., Natick, MA, USA) was used to calculate kinetic parameters. These parameters included the transfer constant, K^trans ^(min^-1^), of gadolinium-based contrast agent between blood plasma and the extravascular extracellular space (EES), and the EES fractional volume, V_e _(%). Parameters were adjusted using the modified Kety model applied to MRI data by both Larsson et al [13] and Tofts and Kermode [34] and fully described recently by various authors such as Buckley [19] or Padhani [7]:

(3) $$C_{t}\left(t\right)=K^{trans}\cdot \int_{0}^{t}C_{p}\left(u\right)\;\cdot \;exp\left(-\frac{K^{trans}}{V_{e}}\cdot \left(t-u\right)\right)\cdot du$$

This model assumes that the plasma volume is negligible. It also assumes that a short bolus injection time is used, with instant mixing and fast exchanges of all mobile protons within the tissue. A pixel by pixel analysis was used given a K^trans ^and a V_e _maps. For all exams, parameters were adjusted twice: with a C_p _obtained directly from the measured AIF (ROIs selecting the internal thoracic artery); with a C_p _related to a calculated theoretical AIF. The theoretical AIF was modeled by using a biexponential decay that corresponds to the results measured by Weinmann et al [27]:

(4) $$C_{p}\left(t\right)=D\cdot \left(a_{1}\cdot e^{-m_{1}\cdot t}+a_{2}\cdot e^{-m_{2}\cdot t}\right)$$

Where D is the injected dose of contrast agent (in millimoles per liter per kilogram of body weight), a_1 _= 3.99 kg/L and a_2 _= 4.78 kg/L are two amplitude constants, m_1 _= 0.144 min^-1 ^and m_2 _= 0.0111 min^-1 ^are two rate constants [27].

### Statistical analysis

The kinetic parameter changes measured after treatment in each group of patients defined by final pathologic findings were compared using non parametric tests (Mann-Whitney U test for two independent random samples and Kruskal-Wallis test for three independent random samples). Kinetic parameter changes of responder patients were compared to the changes of the non-responder patients. Kinetic parameter changes after treatment were compared between complete-, partial-, and non-responder patients. Eventually, the ability of the technique to distinguish the complete responders was tested. All comparisons were performed twice: once with kinetic parameters obtained with measured AIF and then with kinetic parameters estimated with theoretical AIF. The differences in tumour size changes between groups of patients were also compared with nonparametric methods.

Receiver operating characteristic (ROC) analyses of transfer constant and EES fractional volume changes were used to select threshold for the identification of responder and non-responder patients. The diagnostic performance of changes in parameters obtained with a measured or a theoretical AIF were compared by using the approach of DeLong et Clarke-Pearson [35].

Results were analyzed using a statistical software package (Analyse-it Software, Leeds, UK) with an α level set at 5%. All results are given with a 95% confidence interval (95% CI).

Breast tumours size changes in MRI have an accuracy varying from 25% to 93% in detecting tumor response after neoadjuvant chemotherapy [6,29-31]. We estimate that our approach will improve the accuracy from 55% with tumor size changes to 80% using kinetic parameter changes. Thus, sample size of 24 patients was computed to provide 90% power at the overall 5% (two-sided) significance level to detect an accuracy of 80% [32].

## Results

### Patients and Pathologic Response

Patient and tumor characteristics are listed in Table 2. After completion of chemotherapy, within the 24 included patients a Sataloff grade A response was identified in 6 patients, a grade B response was identified in 7 patients, and a grade C+D was identified in 11 patients.

**Table 2:** Patient and Tumor Characteristics (n = 24)

Characteristic	Number of Patients
**Age, years (mean, range)**	48 (range, 31 to 62)
**Tumor histology**	
**Invasive ductal carcinoma**	21
**Invasive lobular carcinoma**	3

**Receptor status**	
**Estrogen, (positive/negative)**	17/4
**Progesterone (positive/negative)**	10/11
**Her2/neu (positive/negative)**	9/12

**Scarff and Bloom Richardson grade**	
**I**	2
**II**	12
**III**	9

**Stage**	
**IIA**	2
**IIB**	5
**IIIA**	7
**IIIB**	8

Using a conventional cutoff value of a decrease of 50% (WHO criteria) of tumor size after treatment compared to baseline, the sensitivity, specificity, positive and negative predictive values, and accuracy of physical examination to detect non-responder were respectively 13% (95% CI:0.8%,54%), 82% (95% CI:48%,97%), 33%, 56% and 53%. The performances of conventional anatomic MRI were 27% (95% CI:6%,61%), 77% (95% CI:46%,95%), 50%, 56% and 54%. Physical examination and conventional morphological MRI based on size measurement were not correlated with the pathologic response after chemotherapy (Mann-Whitney U test, P = 0.44 and P = 0.42 respectively).

Interestingly, physical examination and conventional morphological MRI were more accurate for the detection of complete responders when compared with theirs ability to detect non-responders, (Sensitivity, Specificity, Positive Predictive Value, Negative Predictive Value, and accuracy of 85% (95% CI:54%,97%), 67% (95% CI:24%,94%), 85%, 67%, and 79% with the physical exam and 67% (95% CI:41%,87%), 83% (95% CI:36%,100%), 92%, 45% and 71% respectively in MRI). Physical examination was better correlated with the final pathological response than conventional morphological MRI in detecting complete responders (Mann-Whitney U test, P = 0.05 and P = 0.22 respectively).

### Evaluation of the neoadjuvant chemotherapy in DCE-MRI

The selection and measurement of the internal thoracic artery was possible in all patients (N = 24). Changes in kinetic parameters after the last course of chemotherapy were obtained using measured and theoretical AIF.

#### With measured AIF

**Figure 1:**
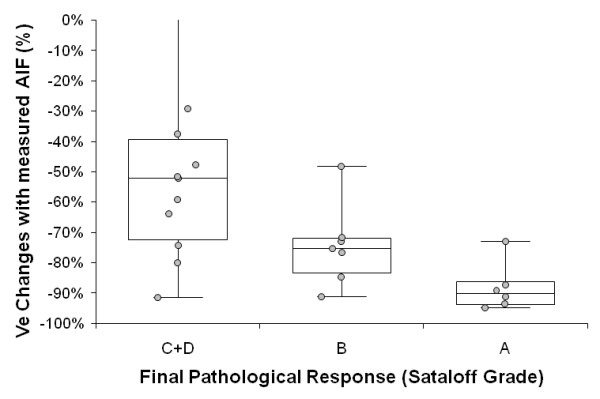
**Changes (median, 1^st ^and 3^rd ^quartile and range) in leakage space (V_e_) after neoadjuvant chemotherapy in complete responders (Sataloff grade A), partial responders (Sataloff grade B) and non-responders (Sataloff grade C+D)**. Kinetic parameters were obtained with measured AIF. The non-responder patient that had an increase in V_e _(+193%) is not shown.

Under treatment, changes in K^trans ^and V_e _were significantly different between non-responders (grade C+D) and responders (grades A+B), (Mann-Whitney U test, P = 0.01, P < 0.01 respectively). Moreover, changes in K^trans ^and V_e _were significantly different between non-responders (grade C+D), partial responders (grade B) and complete responders (grade A) as seen figure 1 (Kruskal-Wallis test, P = 0.04 and P < 0.01 respectively). Eventually, a higher decrease of V_e _(-90% (95% CI:-97%,-80%)) was noted in complete responders (grade A) than in non-complete responders (grade B+C+D), (-68% (95% CI: -82%,-19%); Mann-Whitney U test, P < 0.01). All results are summarized Table 3 and 4.

**Table 3:** Changes in kinetic parameters and tumor size in 24 patients after systemic neoadjuvant chemotherapy

Patient #	K^trans ^changes		V_e _changes		Size changes	Clinical	Final pathologic finding
	AIF theoretical	AIF measured	AIF theoretical	AIF measured	N/A	Response	Sataloff Grade
**1**	-70%	-23%	-73%	-80%	0%	PR	C
**2**	-35%	-20%	-4%	-52%	57%	SD	C
**3**	-2%	108%	-89%	-91%	-100%	PR	C
**4**	-71%	-22%	-46%	-48%	-84%	SD	C
**5**	223%	166%	-76%	-74%	-100%	PR	C
**6**	-97%	-84%	23%	-29%	158%	PR	C
**7**	-85%	-67%	-78%	-64%	-72%	PR	C
**8**	-93%	93%	-15%	-52%	-99%	CR	C
**9**	-98%	-98%	-50%	-38%	-65%	PR	D
**10**	-90%	-82%	-50%	-59%	-100%	PR	C
**11**	-97%	-89%	-49%	193%	-62%	PR	C
**Median (C+D)**	**-85%**	**-23%**	**-50%**	**-52%**	**-72%**		
**95 CI**	**[-110%,17%]**	**[-72%,51%]**	**[-69%,-23%]**	**[-88%,17%]**	**[-98%,13%]**		

**12**	-85%	-95%	-61%	-73%	-71%	CR	B
**13**	-99%	-86%	-65%	-77%	-73%	PR	B
**14**	-54%	-89%	-57%	-48%	-100%	SD	B
**15**	-94%	-95%	-75%	-75%	-33%	SD	B
**16**	65%	-52%	-68%	-72%	-100%	PR	B
**17**	-96%	-84%	-72%	-85%	-91%	PR	B
**18**	-97%	-97%	-77%	-91%	-29%	PR	B
**Median (B)**	**-94%**	**-89%**	**-68%**	**-75%**	**-73%**		
**95 CI**	**[-121%,-10%]**	**[-100%,-71%]**	**[-75%,-61%]**	**[-87%,-62%]**	**[-98%,-44%]**		

**Median (B+C+D)**	**-88%**	**-83%**	**-63%**	**-68%**	**-73%**		
**95 CI**	**[-95%,-14%]**	**[-80%,0%]**	**[-69%,-40%]**	**[-82%,-19%]**	**[-87%,-20%]**		

**19**	0%	27%	-89%	-89%	-40%	PR	A
**20**	-15%	-91%	-62%	-95%	-99%	CR	A
**21**	4%	-54%	-89%	-91%	-100%	CR	A
**22**	-29%	-98%	-51%	-73%	-100%	CR	A
**23**	-99%	-100%	-95%	-94%	-96%	CR	A
**24**	-94%	-99%	-75%	-87%	-97%	PR	A
**Median (A)**	**-22%**	**-94%**	**-82%**	**-90%**	**-98%**		
**95 CI**	**[-87%,10%]**	**[-122%,-16%]**	**[-95%,-59%]**	**[-97%,-80%]**	**[-114%,-64%]**		

Final pathologic findings were graded according to the scale established by Sataloff: complete responder group (grade A), partial responder group (grade B), responder group (A+B), and non-responder group (grade C+ D). Surgery performed after neoadjuvant chemotherapy included mastectomy or conservative treatment or inadequate conservative surgery followed by mastectomy. SD = stable disease, PR = partial responder, CR = complete responder.

**Table 4:** Statistical results (*P-*value) of the non parametrical test (* Kruskal-Wallis and ‡ Mann-Whitney U test) used to compare changes in the kinetic parameters, tumor size in MRI, and clinical findings, between groups of patients defined by final pathologic findings (Sataloff Grade)

	**K**^**trans**^		**V**_**e**_		Size in MRI	Clinical findings
**Sataloff Grade**	**AIF theoretical *p-*value**	**AIF measured *p-*value**	**AIF theoretical *p-*value**	**AIF measured *p-*value**	***p-*value**	***p-*value**

**A vs B vs C+D**	0,5467	0,0398	0,0799	0,0092	0,4643	0,4643
**A+B vs C+D**	0,9095	0,0107	0,0474	0,0059	0,4244	0,4421
***A vs B+C+D***	0,3173	0,1096	0,0532	0,0077	0,2244	0,0462

#### With theoretical AIF

V_e _changes were found to be different between non-responders (grade, C+D) and responders (grades, A+B), (Mann-Whitney U test, P = 0.05). However, no significant difference was found between patient groups with K^trans^.

**Figure 2:**
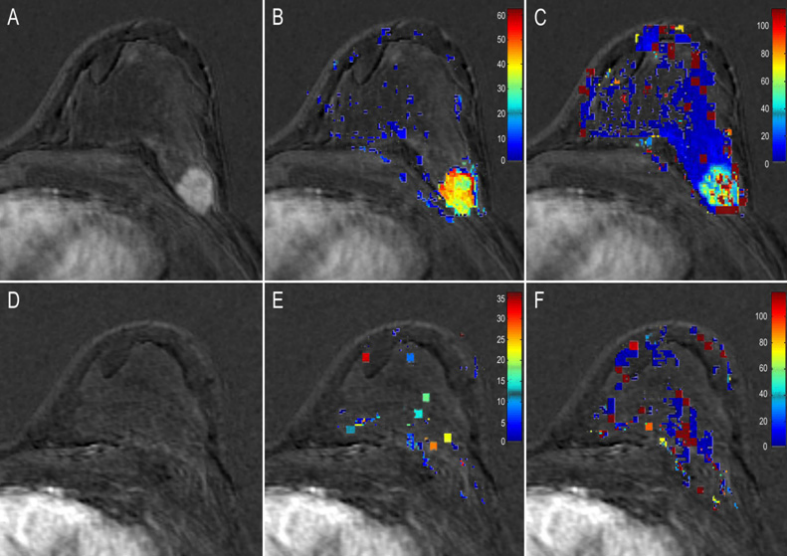
**Images show changes in transfer constant (K^trans^) in patient 22, complete responder to neoadjuvant chemotherapy (Sataloff A)**. Columns show in A and D: anatomic subtraction images; in B and E: corresponding K^trans ^map acquired using measured Arterial Input Function (AIF); and in C and F: corresponding K^trans ^map acquired using theoretical AIF. Images A, B, and C show data before neoadjuvant chemotherapy treatment and images D, F, and G are post-treatment. After treatment a decrease of -98% is seen in K^trans ^using measured AIF values and a decrease of -29% using theoretical AIF values. Note the difference between K^trans ^values before treatment when using measured and theoretical AIFs. To increase visibility of the color encoded K^trans ^pixels the scale was reduced in postchemotherapy images.

**Figure 3:**
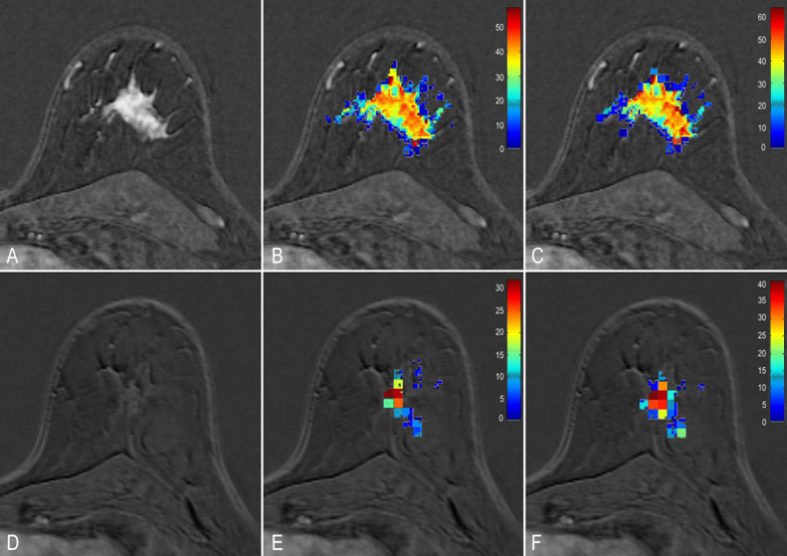
**Images show changes in volume leakage (V_e_) in patient 10 non responder to neoadjuvant chemotherapy (Sataloff grade C)**. Columns show in A and D: anatomic subtraction images; in B and E: corresponding V_e _map acquired using measured Arterial Input Function (AIF); and in C and F: corresponding V_e _map acquired using theoretical AIF. Images A, B, and C show data before neoadjuvant chemotherapy treatment and images D, F, and G are post-treatment. After treatment, low decreases in V_e _median were seen using the measured AIF (-59%) and theoretical AIF (-50%) in agreement with the pathological observation. Note the disagreement with tumour size changes (-100%). To increase visibility of the color encoded V_e _pixels the scale was reduced in postchemotherapy images.

Typical examples of complete responder (#22) and non-responder (#10) patients are given in figure 2 and 3.

#### Identification of responders and non-responders

**Figure 4:**
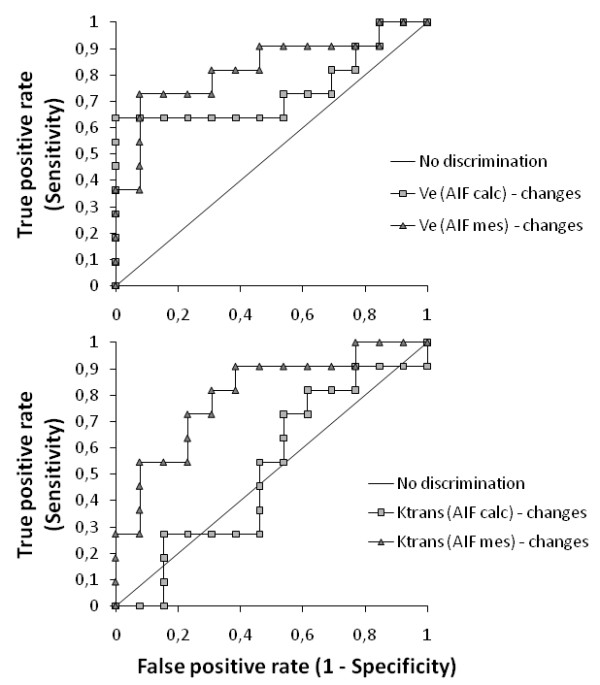
**ROC analysis to differentiate patients' response to neoadjuvant chemotherapy**. Using measured AIF a decrease in V_e _of less than -72% results in 73% sensitivity for identifying non-responders (specificity 92%; area 0.83). Using theoretical AIF, the cutoff value of -51% had lower accuracy (sensitivity 64%; specificity 100%; area 0.74). For transfer constant using measured AIF, a decrease of less than -84% results in 73% sensitivity in the identification of 8 of 11 non-responders patients (Specificity 77%; area under ROC curve 0.80). Using calculated AIF, the cutoff value of -85% had lower accuracy (sensitivity 46%; specificity 54%; area under ROC curve 0.48).

After treatment, a reduction of less than -84% in transfer constant (K^trans^) with measured AIF, would have had a 73% (95% CI:39%,94%) sensitivity in the identification of 8 of 11 non-responders patients (i.e., pathologic subgroup C+D) and would have excluded 3 out of 13 responders (specificity 77% (95% CI:46%,95%), positive predictive value 73%, negative predictive value 77%, accuracy 75%, area under ROC curve 0.80 (95 CI: 0.62,0.99)). With a theoretical AIF to assess K^trans^, the cutoff (-85%) had lower accuracy (sensitivity 46% (95% CI:167%,76%), specificity 54% (95% CI:25%,80%), positive predictive value 46%, negative predictive value 54%, accuracy 50%, area under ROC curve 0.48 (95% CI: 24%,73%)). However, the difference between areas under ROC curves of K^trans ^obtained with measured and theoretical AIF (Figure 4) was not significantly different (Clarke-Pearson test, P = 0.1475).

A reduction of less than -72% in leakage space (V_e_) with measured AIF, would have enabled identification of 8 of eleven non-responders (sensitivity 73% (95% CI:39%,94%)) and would have excluded only 1 out of 13 responders (specificity 92% (95% CI:64%,100%)), positive predictive value 89%, negative predictive value 80%, accuracy 83%, area under ROC curve 0.83 (95% CI: 64%,100%)). With a theoretical AIF to assess V_e_, the cutoff (-51%) had lower accuracy (sensitivity 64% (95% CI:31%,89%), specificity 100% (95% CI:75%,100%), positive predictive value 100%, negative predictive value 77%, accuracy 83%, area under ROC curve 0.74 (95% CI:51%,97%)). However, the difference between areas under ROC curves of V_e _obtained with measured and theoretical AIF (Figure 4) was not significantly different (Clarke-Pearson test, P = 0.3216). Moreover, a reduction of less than -87% in V_e _with measured AIF, would have enabled identification of 5 of 6 complete responders and 16 of 18 non complete responders (sensitivity 89% (95% CI:65%,99%), specificity 83% (95% CI:36%,100%), positive predictive value 94%, negative predictive value 71%, accuracy 88%, area under the ROC curve 87% (95% CI:71%,100%)).

The accuracy of the classification between responders and non-responders was slightly improved when K^trans ^and V_e _were used jointly. With a cutoff of -82% for K^trans ^and -72% for V_e_, all non-responders were distinguished (sensitivity 100% (95% CI:68%,99%)), whereas 4 out of 13 responders were misdiagnosed (specificity 69% (95% CI:39%,90%), positive predictive value 73%, negative predictive value 100%, accuracy 83%).

## Discussion

This clinical study have examined the ability of conventional breast DCE-MRI performed at 1.5T with high spatial resolution (sub millimeter) and low temporal resolution (1 acquisition per minute), to provide changes in kinetic parameters that agree with pathological analysis of response to neoadjuvant chemotherapy. Changes in transfer constant and in leakage space obtained with a measured AIF were significantly different between the three pathologic response categories (Kruskal-Wallis test, P < 0.04). We found that changes in the kinetic parameters correlated pathologic response to neoadjuvant chemotherapy (P < 0.04), whereas both change in MRI-derived tumor size and final clinical examination following neoadjuvant chemotherapy failed to correlate with final pathologic response (Kruskal-Wallis test, P > 0.4), when complete responders and partial responders are considered. It is well recognized that size change is an imperfect assessment method for assessing the effects of neoadjuvant chemotherapy. We found an appreciable discordance between final clinical and final pathologic response in our patient group, with almost a half of clinical responders (13 of 24) failing to obtain a pathologic response. This discrepancy between clinical and pathologic response has been described by others. The NSABP-B18trial [36] showed that of the 682 patients, who received neoadjuvant chemotherapy, 247 achieved a clinical Complete Response but only 88 of these had a pathologic Complete Response.

The microvascular pressures differ from one tumor to another depending upon the vascular architecture, viscous resistance offered to blood flow and interstial fluid diffusion [37-39]. The high interstitial fluid pressure (IFP) seen in tumor without treatment would result in a low diffusion of the contrast agent in the interstitial space and hence low measured V_e _value. In responder, we can assume a decrease of the proportion of immature vessel that yield to a reduction of the IFP and an increase of V_e_. On the other hand, tumor resistance to chemotherapy would result in ongoing production of angiogenic factors that maintain or increase the IFP and V_e_.

We have observed that changes in kinetic parameters obtained in low temporal resolution are more accurate when AIF are measured instead of using a calculated theoretical AIF as usually performed in the literature [20,40]. In this study, the comparison of ROC curves obtained for K^trans ^changes with a measured and a theoretical AIF shows the inability to distinguish responders and non-responder when a theoretical AIF is used. The ROC curves obtained for V_e _changes shows slight improvement in performance to distinguish responders and non-responders, not statistically significant, when measured AIF is used. Wedam et al. have evaluated the effect of Bevacizumab, an antiangiogenic treatment by using the dynamic contrast enhancement MRI with theoretical AIF [41]. The kinetic parameters (K^trans^, k_ep_, and v_e_) were significantly decreased after the first cycle of treatment. However, there was no significant difference in any of the DCE-MRI parameters between clinical responders and non-responders. The use of a calculated theoretical AIF may have reduced the observed decrease in all parameters under treatment and prevented the identification of responders. We suggest that the actual measure of the AIF be used for the calculations of K^trans ^and v_e _when data are obtained in low temporal resolution.

In this study, the selection of the Arterial Input function from the internal thoracic artery was possible in all cases, despite the risk of partial volume artifact due to the small size of the artery [26]. The use of high spatial resolution images helped to identify the internal thoracic artery. Moreover, the low temporal resolution has made possible to manually adjust ROIs on each images to encompass motion artifact and to achieve successfully the AIF in any of the cases. However, computed assisted ROI selections are suitable to achieve results with a lesser time consuming technique [42-44].

Although, the optimal temporal resolution seems to be less than 20 seconds for tracer kinetics modeling [45-47], several kinetic parameters studies with limited temporal resolution obtained with the Tofts model have been proved useful [22,48]. Li et al. have investigated the heterogeneity in the angiogenic response of human breast cancer xenograft to a novel angiogenesis inhibitor. They used the kinetic parameters provided by the Tofts and Kermode model with data obtained in DCE-MRI with a time resolution of 63 seconds. In their study, histogram segmentation showed that changes in the number of voxels within certain segments of the transfer constant histogram were the most sensitive variable for separating control from treated tumors. Planey et al. showed good correlation between K^trans ^and V_e _estimates from data acquired at 16.4-second temporal resolution compared to 33 and 64 second [49]. However, continuous technical improvements observed in MRI, may resolve the dilemma between the diverging demands of high temporal resolution and high spatial resolution. With the advent of new sequences, parallel imaging and the move to higher field strengths 3.0 a temporal resolution of 13 sec with an isotropic voxel size of 1.7 mm is feasible [50]. Also, new multichannel breast coil may help to accelerate sequences allowing better images sampling and faster T1 mapping for an accurate signal conversion into concentrations. In this study, all non-responders were successfully classified by combining K^trans ^and V_e_, whereas the separate analysis had lower sensitivity and specificity. These results incite to obtain more parameters from functional studies, which require faster sequences to accurately predict tumors response to chemotherapy.

It is clear that concentration assessment improves the final accuracy of the kinetic parameters [51]. However, this method requires accurate measurements of the tissue T1 relaxation time before and after contrast injection that is usually performed in few slices due to the temporal resolution constraints [52]. Nevertheless, the effect of the native tissue T1 relaxation on signal enhancement ratio and K^trans^/V_e _is very small in conditions observed in this study: short TR < 10 ms, short TE < T2*, and low dose of Gd-DTPA administration [22,34]. With a temporal resolution of 1.11 minutes, the analysis of the AIF was limited to the decay phase after the first pass, in this study. Concentrations in artery was expected to be low, and a linear relationship between concentration and signal intensity was assumed [25,32]. These assumption seems acceptable since changes in K^trans ^and V_e _achieved with measured AIF allowed to distinguish responders from non-responders (grade, C+D) (Mann-Whitney U test, P < = 0.01). Moreover, changes in V_e _were found helpful to distinguish complete responders from non-complete responders (Mann-Whitney U test, P < 0.01). And hence, a V_e _change threshold may be defined to corroborate breast-conserving surgery in clinical ambiguous patients. All these technical limitations do not allow us to measure the absolute K^trans ^and V_e _values but only relative changes in these kinetic parameters in one tumor between two exams. Though both K^trans ^and V_e _changes could be used to differentiate responders and non-responders, a few non responder patients showed an unexpected large decrease in K^trans^. This could be explained by the antivascular effects of chemotherapy [21,53-56] that would alter the microcirculation parameters (K^trans^) before its effects can be seen in malignant tissue represented by V_e_.

Another limitation of this preliminary study is the small number of patients included. Larger groups of patients can be studied by greater number of radiologists in furthers studies as well as more kinetic parameters with advances in medical imaging (MRI) technology. However, while conventional breast MRI had an accuracy as low as 54%, with a measured AIF we have reached accuracies greater than 80% when a threshold of -72% for V_e _changes or when combined kinetic parameter changes were used in detecting residual breast cancer after neoadjuvant chemotherapy. The inclusion of 24 patients yielded a power greater than 90% to evaluate the response with these accuracies [57]. Recent studies have demonstrated the interest of diffusion and spectroscopy data in the evaluation of breast cancers response to neo adjuvant chemotherapy [58,59]. These parameters could be combined with kinetics parameters [8] and biological data such as hormonal and HER2 receptors status to provide a multimodality comprehensive analysis.

## Conclusion

This study shows that it is feasible to assess tumours' microcirculatory kinetic parameters changes with current breast MRI protocols used in daily clinical practice. These changes in parameters are more accurate when obtained using a measured AIF, and may prove helpful to better determine breast cancer response to neoadjuvant chemotherapy than MRI based on tumor size measurements.

## Abbreviations

ROI: region of interest; K^trans^: inflow rate constant; V_e_: leakage space; DCE-MRI: dynamic contrast-enhanced MRI; MRI: magnetic resonance imaging; AIF: arterial input function; ROC: receiver operating curve characteristic; TE: echo time; TR: repetition time; VEGF: vascular endothelial growth factor; 95 CI: 95% confidence interval.

## Competing interests

The authors declare that they have no competing interests.

## Authors' contributions

The study was conceived by CDB, LF, NS. CDB, CB, DB and RC analyzed the data and contributed to manuscript preparation. CDB, ITN and ASH carried out the MRI experiments. CAC, EDK, OC and ME were involved in drafting the final manuscript.

All authors read and approved the final manuscript.

## Pre-publication history

The pre-publication history for this paper can be accessed here:

http://www.biomedcentral.com/1471-2407/11/361/prepub
